# Effect of Gamma Irradiation on Enhanced Biological Activities of Exopolysaccharide from *Halomonas desertis* G11: Biochemical and Genomic Insights

**DOI:** 10.3390/polym13213798

**Published:** 2021-11-02

**Authors:** Habib Chouchane, Sahar Boutiti, Awatef Ouertani, Wafa Hassen, Sihem Guesmi, Mohamed Neifar, Haikel Jelassi, Haïtham Sghaier, Ahmed Salah Eddine Masmoudi, Ameur Cherif

**Affiliations:** 1 Univ. Manouba, ISBST, BVBGR-LR11ES31, Biotechpole Sidi Thabet, Ariana 2020, Tunisia; saharboutiti@gmail.com (S.B.); awatef.ouertani@gmail.com (A.O.); hassen.wafa@gmail.com (W.H.); guesmisihem152@gmail.com (S.G.); mohamed.naifar@gmail.com (M.N.); tn.cnstn.haitham.sghaier@gmail.com (H.S.); ahmedsleheddine.masmoudi@isbst.uma.tn (A.S.E.M.); ameur.cherif@uma.tn (A.C.); 2 Research Laboratory Energy and Matter for Development of Nuclear Science (LR16CNSTN02), National Center for Nuclear Science and Technology (CNSTN), Sidi Thabet Technopark, Ariana 2020, Tunisia; haikel.jelassi@gmail.com

**Keywords:** antioxidant activities, antitumor activities, gamma-irradiated derivatives, genomic analyses, *Halomonas desertis* G11, native exopolysaccharide

## Abstract

In this work, a native exopolysaccharide (nEPS) produced by *Halomonas desertis* G11 isolated from a Tunisian extreme environment was modified by gamma irradiation. Characterization as well as the antioxidant and antitumor activities of nEPS and its gamma-irradiated derivatives (iEPSs) were comparatively evaluated. In vitro and in vivo antioxidant potentials were determined by using different methods and through different antioxidant enzymes. The antitumor activity was checked against a human colon cancer cell line. Analyses of the complete genome sequence were carried out to identify genes implicated in the production of nEPS. Thus, the genomic biosynthesis pathway and the export mechanism of nEPS were proposed. Analyses of irradiation data showed that iEPSs acquired new functional groups, lower molecular weights, and gained significantly (*p* < 0.05) higher antioxidant and antitumor abilities compared with nEPS. These findings provide a basis for using iEPSs as novel pharmaceutical agents for human therapies.

## 1. Introduction

Exopolysaccharides (EPSs) are carbohydrate polymers synthesized by many organisms such as bacteria, archaea, fungi, and algae [1,2]. They are constituted by a variable number of monomers (typically >10 monomers; oligosaccharides, ≤10 monomers) linked together by glycoside bonds, and therefore, they are characterized by a high molecular weight (MW) [3]. EPSs’ structure can vary from simple (with no branches) to complicated (with different branches); considering their components, they were classified as homopolysaccharides—when composed of one type of monomers—or heteropolysaccharides—when formed by at least two types of monomers. Interestingly, changes in sugar constituents, monosaccharide conformation, alpha and beta binding, non-carbohydrate substituent, charge, and MW led to an immense structural diversity of EPSs [4]. This structural diversity is underpinned by different metabolic pathways, mostly determined by the complement of various EPSs-modifying enzymes available in microorganisms [3]. This structural diversity leads to various biological functions and offers a set of particular physicochemical properties, which could be exploited for various applications. At present, EPSs are used as active components of some biological drugs, dietary supplements, microbial flocculants, emulsifiers, and antioxidants. They are also used as agents to combat aging, radiation exposure, and tumor proliferation [5,6,7,8,9,10,11].

EPSs, as therapeutic biomolecules and mediators in cellular system, have attracted more attention in recent years due to their interesting functional properties [12]. Moreover, they were demonstrated as safe macromolecules with minimal adverse effects and could be used for food and pharmaceutical applications [13]. Strikingly, microbial EPS (mEPS) have attracted the attention of nutritionists and scientists because of their several biological capacities, such as antifatigue, antioxidant, antitumoral, anti-inflammatory, hepatoprotective, and immunomodulationa ctivities [9,11,14,15,16,17]. Moreover, experts in the field recognized that the chemical structure of EPSs can be modified by suitable processes in order to strengthen their functional properties. During the last few years, various methods and techniques of EPSs structural modifications have been investigated. These strategies involve (i) chemical derivatizations such as sulfonylation, carboxymethylation, phosphorylation, acetylation, and selenylation [7,9,18,19,20]; (ii) physical methods such as ultrasonic and gamma radiation [9,20], (iii) in vivo modifications based on enzymatic transformations coded by genes involved in EPSs production [21,22]. Previous research established that these methods enhanced the biological activities of EPSs [23,24,25]. Sulfated modification by sulfonation of an EPS from *Lactobacillus Plantarum* ZDY2013 has significantly increased its antioxidant activity, and the sulfated EPS was more effective in countering enterotoxin-induced cytotoxicity on Caco-2 cells [10]. Similarly, Wang et al. [26] demonstrated that a modified EPS—obtained by acetylation, phosphorylation, and carboxymethylation of EPS produced by *Lactobacillus Plantarum* 70810—exhibited stronger antioxidant and antitumor activities. Moreover, Qin et al. [6] demonstrated that the esterification ratio on the degree of sulfate substitution of the EPS produced by *Rhizopus nigricans* leads to stronger inhibitory effects on human colon cancer HCT cells. Furthermore, a sulfated EPS derivative obtained by the sulfonation of native neutral EPSs GX1-1 produced by *Penicillium* sp. significantly promoted the pinocytic activity of RAW264.7 cells [27]. In the same vein, EPS named PLEP-2b and SLEP-2b obtained by phosphorylated and sulfated modifications of LFP-2b polysaccharide produced by *Lachnum* sp. showed an increase in antioxidant activities against hydroxyl radical and superoxide anion as well as remarkable anti-proliferative effects against CT-26 murine colon carcinoma cells [28]. An ultrasound method was applied by Du et al. [20] to modify the physicochemical properties of an EPS produced by *Schizophyllum commune*. The treatment decreased the MW and the viscosity of the EPS and enhanced its anti-inflammatory activity compared with native EPS. A novel EPS from *Lachnum* YM281 was modified with selenium, and its antihyperlipidemic and hepatoprotective properties were evaluated. Results showed that the modified EPS exhibited more protective effects against fatty liver than the native EPS by restoring the histopathological status of liver tissues in a hyperlipidemic mouse model [7].

Although these methods can achieve the desired structural changes and reduce the MW of the EPS, they have some drawbacks related to treatment time, financial cost, and post-processing produced acidic residues [29,30]. Therefore, it is advisable to apply methods that are fast, efficient, and convenient in modifying the nEPS in order to obtain the expected level of the desired bioactivity.

Radiation treatment could be a clean and additive-free alternative for obtaining new materials with high added value based on renewable EPSs [9,30,31]. This method is also considered as an energy-saving technology. Moreover, its use in the sterilization of final biological products has demonstrated their depolymerization by the cleavage of glycosidic linkages, thus modifying their chemical properties [32].

This work was designed to untangle the characteristics, including the antioxidant, and anti-proliferative capacities, of the nEPS of *H. desertis* and its gamma-irradiated derivatives and to identify, for the first time, through genomic analyses, the underlying mechanisms of its biosynthesis and export.

## 2. Materials and Methods

### 2.1. Cell Growth, nEPS Production, and Gamma Irradiation

The strain used in this study was *Halomonas* sp. G11 previously isolated from an extreme Tunisian environment Chott El-Djerid (N 33°59′558″, E 08°39′212″) and renamed *H. desertis* G11 based on genomic and phylogenetic analyses [33,34]. The genome shotgun project of G11 has been deposited at DDBJ/ENA/GenBank under the accession LYXG00000000.

G11 was cultured in a modified MY medium containing 10% *w*/*v* marine salt, glucose concentration of 3% *w*/*v* at 30 °C and an initial pH of 7.0. nEPS synthesis was evaluated using the protocol described in our previous works [9,14].

*H. desertis* G11 growth and nEPS synthesis were determined simultaneously by measuring the optical density at 600 nm using a spectrophotometer (Analytic Jena SPEKOL 2000) and weighing the lyophilized nEPS. Samples of lyophilized nEPS were irradiated by gamma rays emitted by a Cobalt 60 source at doses of 1 and 1.5 kGy with 37.5 Gy min^−1^ dose rate.

### 2.2. Structural Characterization of Native EPS and Its Gamma-Irradiated Derivatives

The carbohydrate content, in the purified nEPS, was evaluated by the method of Chaplin and Kennedy [35]. First, 210^−1^mL of nEPS and phenol solution (5%, *w/v*) were mixed, to which 1 mL of concentrated H_2_SO_4_ was added. The carbohydrate amount in nEPS was estimated by reading the absorbance at 49.2 nm of the obtained solution against the glucose standard curve used as a standard.

The amount of uronic acid in nEPS was checked according to the protocol of Blumenkrantz and Asboe-Hansen [36] with modifications [14]. nEPS (200 μL) was added to 1.2 mL of sodium tetraborate solution (1.25 × 10^−2^ M) in concentrated sulfuric acid, vortexed, and then incubated at 100 °C. Then, 20 μL of metahydroxydiphenyl (0.15%) in NaOH (0.5%) was added to the mixture.To overcome interference with neutral sugars, nEPS was treated with sulfamate before H_2_SO_4_ hydrolysis. Uronic acid content was evaluated using a glucuronic acid standard curve. Absorbance was measured at 520 nm after 30 min incubation.

The sulfate content of nEPS was determined using the protocol of Dodgson and Price [37] after hydrochloric acid hydrolysis (5 mg EPS + 0.2 mL HCl, 1 M for 6 h at 110 °C). The obtained hydrolysate was added to a solution of trichloroacetic acid (3.8 mL, 3.0% *w*/*v*) and barium chloride–gelatin (1.0 mL) and well vortexed. The sulfate content in nEPS was estimated by using a K_2_SO_4_ standard curve. The absorbance was measured at 360 nm after 15 min of incubation using HCl as a blank.

The monosaccharide content of nEPS was obtained by an HPLC-RID (Agilent Technologies 1200 series) according to the method already described in our previous research [14].

MWs of the purified nEPS and its gamma-irradiated derivatives were evaluated by gel column chromatography with a Sephadex G-100 [38]. MWs were estimated by plotting a calibration curve using different dextran standards (7, 10, 50, and 70 kDa).

Morphological analysis of nEPS and gamma-irradiated derivatives were performed by SEM; samples were treated as described in our previous work [14]. Firstly, they were fixed with 5% glutaraldehyde solution for 90 min at 25 °C in a closed Falcon tube. Then, samples were gradually dehydrated with acetone at different concentrations (40%, 50%, 80%, and 100%) for 15 min and coated with gold and observed by a Leo 435 VP SEM (Germany, SRV-32 software) at 15 kV.

Substituting and functional groups were determined by FTIR spectroscopy analyses. The samples of nEPS or gamma-irradiated derivatives (1 mg) were added to 100 mg KBr powder and pressed into pellets (16 mm diameter mold) for FTIR analysis (4 cm^−1^ of resolution; 400–4000 cm^−1^ wave number range). The FTIR spectra were measured by a spectrophotometer (Bruker Vertex 70 FTIR spectrometer).

### 2.3. Biological Characterization of Native EPS and Its Gamma-Irradiated Derivatives

#### 2.3.1. In Vitro Antioxidant Activities

In vitro antioxidant potentialities of native and irradiated (1 and 1.5 kGy) EPSs samples were evaluated using three different methods: 1,1-diphenyl-2-picrylhydrazyl (DPPH), 2,2′-azino-bis-3-ethylbenzthiazoline-6-sulphonic acid (ABTS), and ferric-reducing antioxidant power (FRAP). Butylated hydroxytoluene (BHT) was used as a standard, and experiments were replicated and averaged.

##### DPPH Radical Scavenging Activity (RSA)

The nEPS and iEPSs-RSA of DPPH were evaluated as described by Zhang et al. [39]. Different concentrations of nEPS or iEPSs (10–200 µg mL^−1^) were separately added to 2 mL of DPPH (0.2 mM), well vortexed, and incubated at 25 °C for 30 min. Formula (1) was applied to estimate the inhibition rate of DDPH radicals (DPPH IR %).

(1) $$\mathrm{DPPH}\;\mathrm{IR}\;\%=\frac{1-\left(I_{i}–I_{j}\right)}{I_{0}}\;\times \;100\%$$

where *I_i_* is the absorbance of the nEPS or iEPSs with DPPH, *I_j_* is the absorbance of the nEPS or iEPSs free of DPPH, and *I*_0_ is the absorbance of pure DPPH at 517 nm.

##### ABTS Scavenging Capacity (ABTS-SC)

The ABTS-SC was evaluated according to the protocol of Re et al. [40]. Firstly, the ABTS free radical was oxidized to ABTS* by K_2_S_2_O_8_. The ABTS* solution was diluted with methanol (pH 7) until the absorbance of 0.7 at 730 nm. Samples of nEPS and iEPSs were dissolved in distilled water. Then, 100 µL of each nEPS and iEPSs samples were added to 3.9 mL of the diluted ABTS^*+^ and incubated for 20 min. Finally, absorbencies at 730 nm were determined using methanol as a control. The ABTS-SC percentage was estimated by Formula (2).

(2) $$\mathrm{ABTS}\;\mathrm{SC}\;\%\;=\frac{Ab_{730,C}–Ab_{730,S}}{Ab_{730,C}}\times 100$$

where *Ab*_730*,C*_ is the control absorbance and *Ab*_730*,S*_ is the different samples’ absorbencies.

##### The Ferric-Reducing Antioxidant Power (FRAP)

The protocol of Benzie and Strain [41] was applied to evaluate the antioxidant capacities of nEPS and iEPSs by the FRAP assay. The protocol is based on the potential of nEPS and iEPSs to reduce Fe^3+^ to Fe^2+^ with 2,4,6-tripyridyl-s-triazine (TPTZ) as a complexing reagent. AFRAP solution of 3 M of acetate buffer at pH 3.6 contained TPTZ, HCl, and FeCl_3_6(H_2_O) at molarities of 0.1, 0.4, and 0.2 M, respectively, was prepared. Then, 1mL of each concentration of nEPS or iEPSs was added to 5 mL of the FRAP solution and incubated for10 min at 37 °C. Absorbencies of different solutions were measured at 590 nm. Different solutions (from 0.05 to 0.3 mM) of FeSO_4_ were used, in the same condition of nEPS or iEPSs samples, to build a calibration curve and evaluate the antioxidant power of different samples.

#### 2.3.2. In Vivo Antioxidant Activities

##### Experimental Design

In this protocol, 50 male albino mice with body weights ranging from 22 to 25 g were housed for 10 days at room temperature at 40–50% relative humidity, exposed to a 12 h light/dark cycle, and fed with high-fat (HF) diet to induce oxidative stress. The HF diet was composed of normal food supplemented with beef tallow and condensed milk. The HF diet contained approximately more than 50% fat in terms of caloric content. After the acclimatization period, mice were randomly divided into five groups and then used in the experimental protocol as described in Table 1.

##### Biochemical Assay

For biochemical analyses, mice were anesthetized and sacrificed after treatment period on day 15 (Table 1). Blood was collected under standard conditions and centrifugated at 3000× *g* for 20 min at 4 °C. The obtained serum was stored at −80 °C for subsequent analyses. The antioxidant enzymes CAT, SOD, and GSH-Px activities and the content of the oxidative damage indicator MDA in each group were evaluated using different Abcam kits (ab83464), (ab65354), (ab102530), and (ab118970), respectively, according to the manufacturer’s instructions.

### 2.4. Antitumor Activities

The anti-proliferative activities of nEPS from *H. desertis* and its gamma-irradiated derivatives iEPSs (1.0 and 1.5 kGy) were evaluated against colon cancer cells (HT-29 cell line). Colon cancer cells were cultured in DMEM medium enriched with 10% (*v*/*v*) fetal bovine serum and supplied with penicillin and streptomycin (100 UmL^−1^). The cell culture was incubated at 37 °C in a 5% CO_2_ atmosphere. The HT-29 cell lines were treated, at different incubation periods (24 and 48 h), with different doses of nEPS and iEPSs (0, 50, 100, 150, 200, and 250 µg mL^−1^). Fluorouracil (5-FU, 50 µg mL^−1^) was used as a positive control. The protocol using MTT assay developed by Chen et al. [42] was applied to evaluate the anti-proliferative activities of nEPS and iEPSs against colon cancer cells.

### 2.5. Structural Genomic Analyses

Previous literature has shown that despite the great structural diversity, bacteria produce EPSs through two main processes called sequential and en bloc mechanisms via four different pathways: (i) the extracellular production using glycosyltransferase, (ii) the synthase-dependent pathway, (iii) the ATP-binding ABC transporter pathway, and (iv) the Wzy-dependent pathway. Only the last one was characterized as an en bloc mechanism [3,12]. It should be mentioned that a given bacterial species could harbor one or more pathways to produce different EPSs. These mechanisms are well studied mainly in lactic bacteria (LAB) [3]. However, it is important to point out that from a genetic point of view of *Halomonas* bacteria information related to the EPS biosynthesis, particularly genes involved in the polymerization of repeat units and EPS transportation out of the cell are scanty and not yet understood [43]. Consequently, genes implicated in nEPS production were identified within the genome of *H. derstis* G11 using the RAST server [44]. All identified proteins were blasted against the UniProt database (https://www.uniprot.org/, accessed on 24 March 2021) to confirm their functions.

### 2.6. Statistical Analyses

Data were computed as means of three replicates of three independent trials. The statistical analysis of the obtained results was performed using one-way analysis of variance (ANOVA). Multiple comparisons were performed to assess differences between groups. A *p* value < 0.05 was maintained as statistically significant.

## 3. Results and Discussion

### 3.1. Cell Growth and nEPS Synthesis

**Figure 1:**
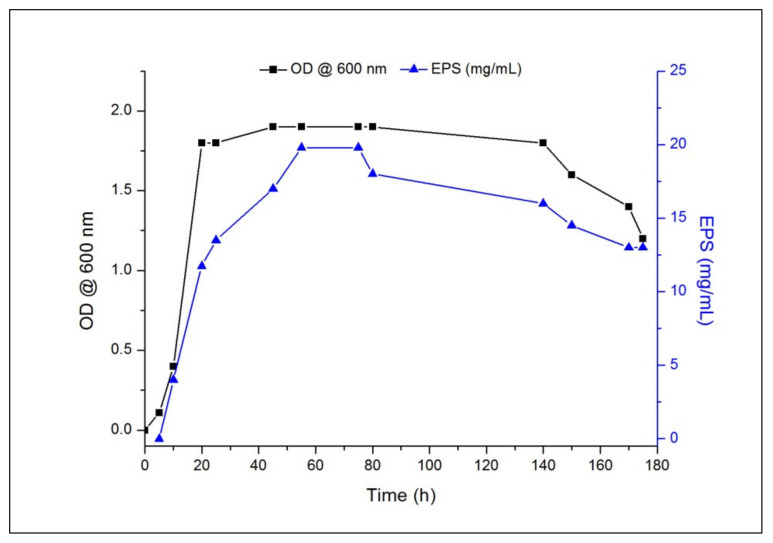
Profile of growth and native exopolysaccharide (nEPS) production by *H. desertis* in modified MY medium (10% *w*/*v* salts, 3% *w*/*v* glucose) at 30 °C. (■), optical density @ 600 nm; (▲), mg EPS per mL of culture medium.

As shown in Figure 1, halotolerant bacterium *H. desertis* G11 is able to grow and synthesize nEPS for seven days at 30 °C on a modified MY medium containing 10% *w/v* marine salt and 3% *w/v* glucose. Under these conditions, maximum growth (OD_600_ = 1.9) was achieved in 45 h. What is interesting about the data in Figure 1 is the synthesis of 20 g L^−1^ of nEPS after 58 h based on the metabolism of the carbon. nEPS production kinetics demonstrated that the polymer was mostly released during the period of exponential growth, and then, the rate of nEPS production remained relatively constant (above 15 g L^−1^) in the time interval from 40 to 140 h (stationary growth period), and further incubation (exceeding 140 h) resulted in the decrease in the amount of nEPS (below 15 g L^−1^). Based on previous works [45,46,47], the maximum yield of EPS production by *Halomonas* strains was always observed during the exponential growth phase. Compared to other EPS-producing *Halomonas* strains, the yield of EPS produced by *H. desertis* was 12-fold higher than that of *H. almeriensis* (1.7 g L^−1^) [45]; 5-fold higher than that of *H.* Maura (3.8 g L^−1^) [46]; and 4-fold higher than that of *H. anticariensis* (5 g L^−1^) [47].

### 3.2. Structural Characterization of nEPS and Its Gamma-Irradiated Derivatives

The results of the calibration curve (OD = f ([glucose])), used to determine the carbohydrate content in nEPS, are provided in Figure S1A (Supplementary data). The absorbance of the nEPS hydrolysate was 1.0; from the calibration curve, this absorbance value gives a concentration of 1.22 mg mL^−1^ (glucose equivalent). Considering the initial used concentration of nEPS (2 mg mL^−1^), the total carbohydrate content was equal to 61%. By following the same steps and the corresponding calibration curves (Figure S1B,C, Supplementary data), the amount of uronic acid and sulfate were estimated to 17.5% and 10.2%, with ODs = 2.63 and 0.02, respectively.

Additionally, the purified nEPS did not contain proteins or nucleic acids. Indeed, nEPS showed a negative response to the Bradford’s test and did not absorb at 260 and 280 nm. Compared to other *Halomonas* strains, nEPS exhibited distinctive chemical features including high levels of uronic acid and sulfate (Table 2). The sulfate content of the nEPS is especially interesting. Indeed, EPSs endowed with interesting biomedical properties contain sulfate in their composition, and this appears to be an essential characteristic with respect to their activities [14,48].

**Figure 2:**
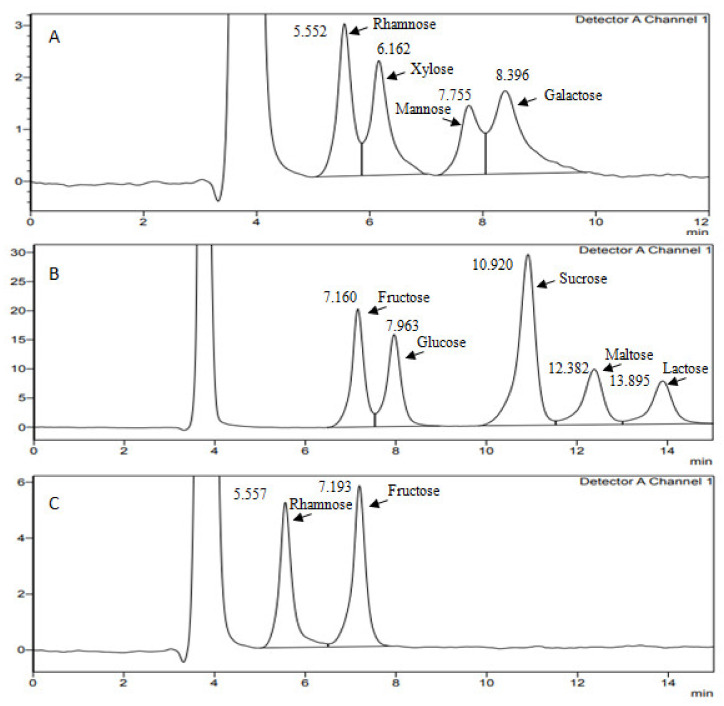
HPLC-RID chromatogram of (**A**,**B**) standard monosaccharides (rhamnose, xylose, mannose, glucose, galactose, fructose, sucrose, maltose, lactose) and (**C**) nEPS hydrolysate from *H. desertis* G11.

From the HPLC-RID data in Figure 2, it is apparent that nEPS produced by the strain G11 is an heteropolysaccharide containing two monomers, rhamnose and fructose. The most interesting aspect of Figure 2 is the detection of fructose, for the first time to the best of our knowledge, in the composition of EPSs produced by *Halomonas* strains; contrarily, rhamnose is present in the composition of EPSs synthesized by different strains of *Halomonas* [45,50,52].

**Figure 3:**
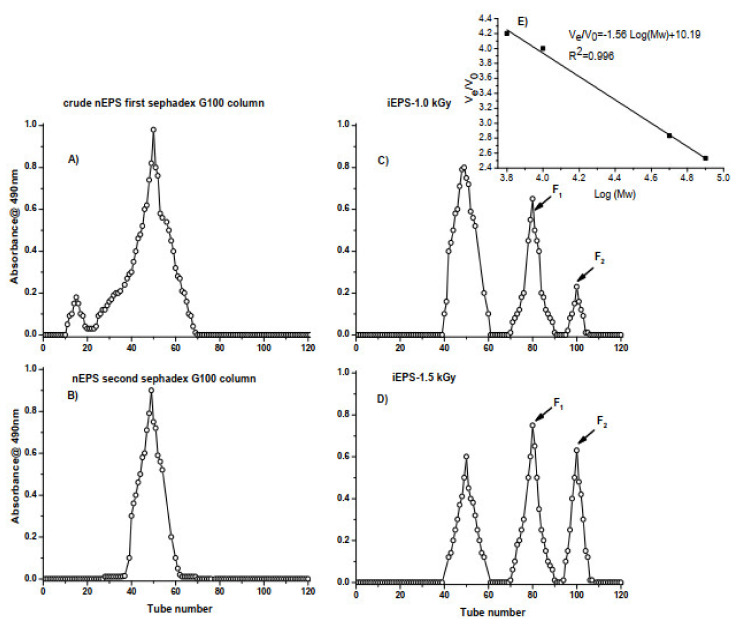
Elution curves of crude nEPS (**A**), purified nEPS (**B**), and gamma-irradiated derivatives iEPS 1 kGy (**C**) and iEPS 1.5 kGy (**D**) by Sephadex G-100 gel filtration. The volume of each fraction was 2 mL, and the eluates were checked by measuring the absorbance at 490 nm, (**E**) standard curve of the relative molecular weight (MW).

The MW of EPSs is considered among the crucial factors involved in their biological properties [54]. The weight average MW of nEPS and iEPSs was determined to be 2.42 × 10^4^ Da, 1.27 × 10^3^, and 1.78 × 10^2^ Da, respectively (Figure 3A–D), according to the calibration curve V_e_/V_0_ = f(log MW), plotted with standard dextrans, the formula V_e_/V_0_ = −1.5624 Log (MW) + 10.186 and R^2^ = 0.99, as shown in Figure 3E. As shown in Table 2, the MW of the present nEPS was comparable to those synthesized by *H. anticarinesis* FP36 [47], *H. almeriensis* M8^T^ [45], and *H. saliphila* LCB169^T^ [53] and lower than the MW of EPS produced by *Halomonas* sp. MCTG39a [51] and *H. smyrnensis* K2 [52]. Strikingly, both iEPSs-containing fragments F1 and F2 (Figure 3) obtained by gamma irradiation with 1.0 and 1.5 kGy displayed the lowest MW. Previous reports have pointed out that EPS with high MW improves viscosity, while those with low MW have a distinctive role as bioactive materials [54,55].

**Figure 4:**
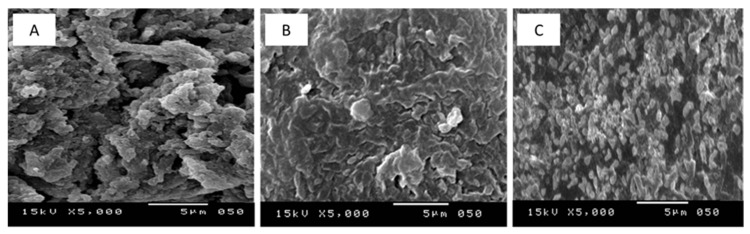
SEM micrographs of (**A**) native exopolysaccharide nEPS, (**B**) gamma-irradiated exopolysaccharide at 1.0 kGy: iEPS–1.0 kGy, (**C**) gamma-irradiated exopolysaccharide at 1.5 kGy: iEPS–1.5 kGy, SEM operating at an accelerating voltage of 15 kV.

As can be seen from the SEM image in Figure 4A, the shape of the nEPS is rugged, damp, and karstified with pits and a few small openings on the surface. Similar SEM micrographs were previously reported for hetero-EPSs produced by extremophilic microorganisms [8,9]. Comparative analyses, conducted by the program Image J [56], of nEPS and iEPS SEM micrographs (Figure 4A–C) demonstrated a significant decrease in Feret’s statistical diameter from 0.284 to 0.122 and significant increase in the total area from 4.825 to 21.742 (*p* < 0.05) due to gamma irradiation effects. Moreover, gamma irradiation caused a reduction in the MW of nEPS in comparison with iEPS (Figure 3). A previous work [9] stated that the exposure of *Bacillus siamensis* CV5 EPS to 0.5, 1, and 1.5 kGy caused its fragmentation into smaller granules. Moreover, Kaur et al. [57] reported that the irradiation of β-glucan modified its primary structures and spatial conformation. Taken together, these results suggested that gamma irradiation could boost the bioactivity of EPS through the acquisition of new structural characteristics, a reduction in the Feret’s diameter and the MW, together with an improvement in the global area.

**Figure 5:**
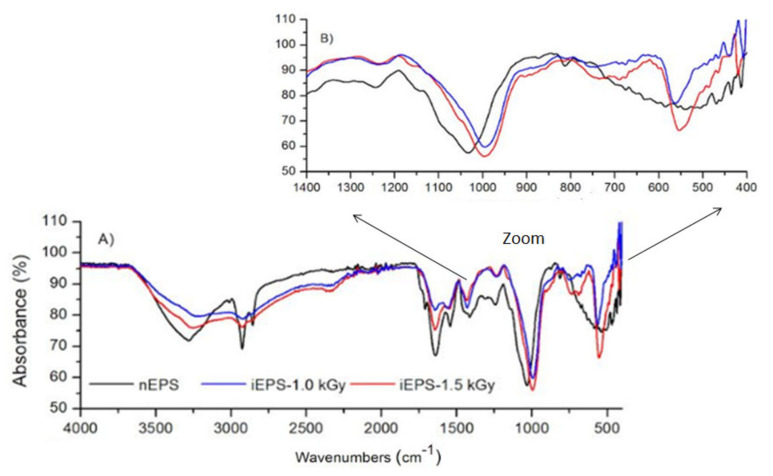
FTIR spectra of (**A**) native exopolysaccharide nEPS, gamma-irradiated exopolysaccharide iEPS–1.0 and iEPS–1.5 kGy over a wavenumber range of 400–4000 cm^−1^, and (**B**) fingerprint region with peaks in the 1400 to 400 cm^−1^ range.

FTIR was used to detect specific groups, to characterize covalent bonding information, and to identify the type of glycosidic linkage in nEPS. In addition, the FTIR spectrum of nEPS was compared to those observed in FTIR spectra of iEPS to pinpoint other possible modifications caused by gamma-irradiation (Figure 5). In Figure 5A, the absorption band in the area around 3750 cm^−1^ was assigned to the hydroxyl stretching vibration of nEPS, which is the characteristic absorption band of the carbohydrate ring. The bands obtained from 3000 to 2900 cm^−1^ are related to the CH of the methyl and methylene groups CH2, which are features of fructose and rhamnose. Bands detected at 2650 cm^−1^ were assigned to the hydroxyl OH of carboxylic acids. The large peak observed at 1750 cm^−1^ was attributed to C=O carbonyl group of esters. The surface of this stretching absorption suggested that nEPS contains a significant amount of acetate and pyruvate, which is in accordance with chemical analyses. The peak at 1500 cm^−1^ was ascribed to carboxylate groups (-COO). The area between 1400 and 1200 cm^−1^ is indicative of sulfate substitution in nEPS. The band observed at 1240 cm^−1^ could be attributed to the O=S=O stretching vibration of sulfate ester. Additionally, the peak at 1200 cm^−1^ suggested the S=O stretching vibration of sulfate groups in nEPS. These findings are in line with the chemical analysis of the sulfate content of nEPS. The absorption band around 1050–1000 cm^−1^ was assigned to C-O-C and C-O glycosidic bands. It is important to note that there is no vibration in the region of 890 cm^−1^, which indicates that nEPS does not include β-glycosidic linkage. Interestingly, the presence of an absorption band around 800 cm^−1^ argues in favor of an α-glycosidic bond between rhamnose and fructose monomers in nEPS.

The comparison between nEPS FTIR spectra with those of iEPSs (Figure 5) clearly illustrates the changes caused by gamma irradiation. Indeed, some absorption peaks observed in the nEPS were either shifted or disappeared after irradiation experiments. For instance, we noted the disappearance of picks in the area from 3000 to 2800 cm^−1^ and at 1750 cm^−1^. Additionally, characteristic variations were significantly remarkable in the fingerprint region (1400 to 400 cm^−1^) of iEPSs FTIR spectra (Figure 5B). Changes in the absorbance intensity in the 800–400 cm^−1^ region with the presence of a new band at 550 cm^−1^ in iEPSs FTIR spectra were also detected. We particularly noted a drop in the intensity of the characteristic signal 800 cm^−1^ corresponding to α-glycosidic linkages. The most striking result to emerge from these data is the destruction of alpha linkages and the appearance of fragments F1 and F2, with low MW, after nEPS irradiation (Figure 3).

### 3.3. Biological Characterization of nEPS and Its Gamma-Irradiated Derivatives

#### 3.3.1. In Vitro Antioxidant Activities

**Figure 6:**
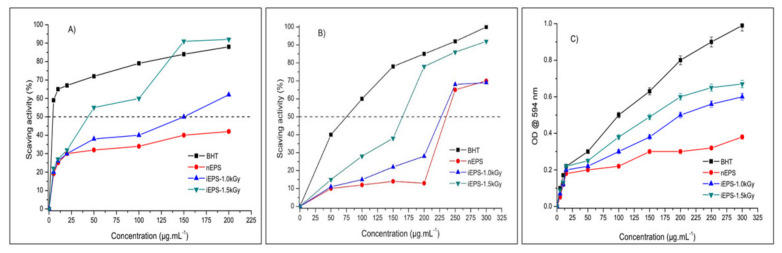
In vitro antioxidant activities of native exopolysaccharide (nEPS) and gamma-irradiated exopolysaccharide iEPS–1.0 and iEPS–1.5 kGy produced by *H. desertis* (**A**) DPPH scavenging activity, (**B**) ABTS scavenging activity, and (**C**) FRAP-Fe^3+^ reducing power. BHT was used as a reference.

The in vitro antioxidant abilities of nEPS and iEPSs were investigated through three different methods, DPPH, ABTS, and FRAP, using BHT as a reference. The findings are presented in Figure 6. The DPPH RSA of the nEPS and iEPSs are reported in Figure 6A; for concentrations below 22 µg mL^−1^, the antioxidant activities showed no difference and remained relatively the same. The DPPH RSA increased from 22 to 200 µg mL^−1^ for all EPSs samples. However, the activity of iEPSs is much higher than that of nEPS, which may be explained by their capacity to donate hydrogen, leading to the formation of stable DPPH-H molecules. In fact, the IC50 of DPPH radical scavenging for the nEPS, iEPS–1.0, and iEPS–1.5 kGy was 200, 150, and 43.75 µg mL^−1^, respectively. Indeed, iEPS–1.5 kGy antioxidant activity, based on its IC50, increases almost five-fold when compared to that of nEPS. Moreover, we noticed that for concentrations above 150 µg mL^−1^, the scavenging rate of iEPS–1.5 kGy becomes more important than that of BHT. A 150 µg mL^−1^ of iEPS–1.5 could scavenge DPPH radicals with an elimination rate of 95%. The nEPS and iEPSs DPPH scavenging activities were better than that of CS-EPS (IC50 = 2.5 mg mL^−1^) from *Halogeometricum borinquense* [14]. They were also more active than EPS-lr and EPS-lvg polymers from *Lactobacillus reuteri* SHA101 and *Lactobacillus vaginalis* SHA110 with scavenging abilities of 78.7 and 70.3 at 4 mg mL^−1^, respectively [58].

As shown in Figure 6B, related to the scavenging capacities of ABTS radicals by nEPS and iEPSs, all tested EPSs demonstrated capacities to bind O_2_^−^ in a concentration-dependent manner. Therefore, the scavenging rate of nEPS, iEPS–1.0, and iEPS–1.5 kGy was significantly elevated when the concentration of EPSs increases from 10 to 200 µg mL^−1^. The IC50 of ABTS radical scavenging for nEPS, iEPS–1.0, and iEPS–1.5 kGy was 237.5, 225, and 162.5 µg mL^−1^, respectively. Considering the IC50, the iEPS–1.5 kGy antioxidant activity was enhanced one and a half-fold compared to nEPS. Both nEPS and iEPSs from *H. desertis* were more effective than the native EPS and its gamma-irradiated derivatives (20 ≤ IC50 ≤ 30 µg µL^−1^) from *B. siamensis* [9].

In addition, the FRAP-Fe^3+^-reducing capacities of nEPS and iEPSs samples increased as the G11 EPS concentration changed from 10 to 200 µg mL^−1^ (Figure 6C). A dose–response relationship was observed between different concentrations of EPS and FRAP-Fe3^+^-reducing powers. Notably, using the same concentration, the reducing power of iEPS–1.0 and iEPS–1.5 kGy was higher than that of nEPS. Moreover, the reducing power of iEPS–1.5 kGy increased from 0.3 to 0.7, which was the most obtained reducing ability with doubled effectiveness when compared to nEPS.

Together, these results related to different in vitro antioxidant assays provide important insights into the interesting antioxidant potentials of G11 iEPSs compared to nEPS (Figure 6). Firstly, the gamma-irradiation of nEPS enhanced the levels of scavenging rates. Since the scavenging activities were mostly explained by the ability to release hydrogen by EPSs [59], our findings suggested that gamma-irradiation boosts the donating capacities of iEPSs and thus reacted with free radicals more than the nEPS. The enhanced scavenging activities of iEPS are due to the breaks, by irradiation to low MW fragments (fragments F1 and F2), as well as to physicochemical changes of the nEPS as suggested by gel permeation chromatography (Figure 3) and SEM micrographs (Figure 4). The breakdown of nEPS leads to an increase in the availability of hydroxyl groups and most importantly to the creation of new exposed functional groups in iEPSs, as clearly proven by FTIR analyses (Figure 5), which inevitably resulted in an increase in antioxidant capacities. In agreement with our results, Choi et al. [60] indicated that gamma irradiation caused the depolymerization of polysaccharides from *Tamarindus indica* seeds. Moreover, a previous study has shown that low MW polysaccharides have more reducing ends, which allows them to scavenge more free radicals, thus enhancing their antioxidant abilities [61]. Similarly, the breakdown of G11 nEPS by gamma-irradiation could enhance the number of reducing ends in iEPSs.

#### 3.3.2. In Vivo Antioxidant Activities

**Figure 7:**
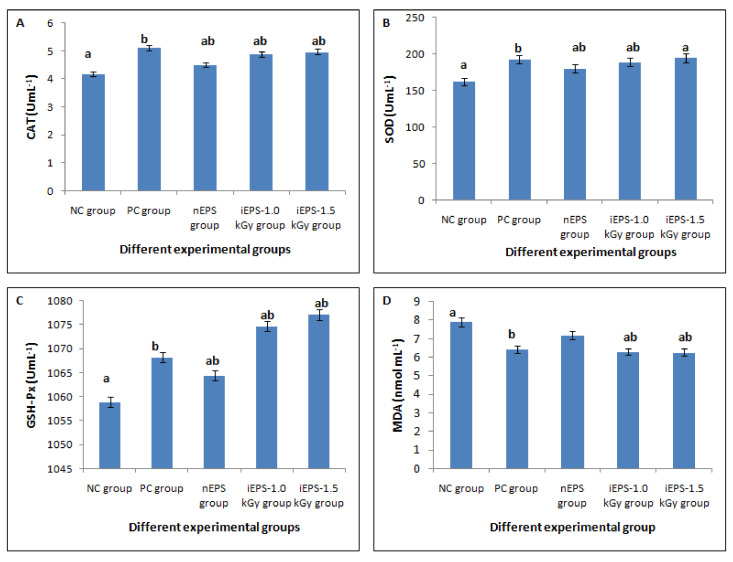
Effects of nEPS and iEPSs on the levels of antioxidant enzymes (**A**) CAT, (**B**) SOD, (**C**) GSH -PX, and (**D**) MDA content in mice. a–b with different letters means significantly different at *p* < 0.05.

The five groups of mice were fed with an HF diet and orally administrated with saline solution, ascorbic acid, nEPS, iEPS–1.0 kGy, and iEPS–1.5 kGy, as indicated in Table 1. After the feeding trial, serum was separated, and the antioxidant enzymes (CAT, SOD, GSH-Px) activities and the amount of MDA in each group were determined to understand the effects of nEPS and iEPSs administration on HF diet-induced oxidative stress. As shown in Figure 7, after dietary supplementation with nEPS, a significant increase in CAT, SOD, and GSH-Px levels and a decrease in the level of MDA were obtained. However, the level of MDA has no significant difference when compared to the NC group (*p* < 0.05) (Figure 7D). Interestingly, the antioxidant status of iEPS–1.0 and iEPS–1.5 kGy groups was more improved by iEPSs administrations than that of the nEPS group. In fact, significant enhancements are achieved with increased CAT, SOD, and GSH-Px levels (*p* < 0.01) and a significant decrease in MDA level (*p* < 0.01). These improvements are close to those observed in the acid ascorbic PC group for CAT with 4.89 UmL^−1^ and 4.97 UmL^−1^ for iEPS–1.0 kGy and iEPS–1.5 kGy vs. 5.12 UmL^−1^ for the acid ascorbic PC group (Figure 7A). It also can be seen that the SOD activities in the treatment with iEPSs considerably increased compared with the NC group and exceeded that of the acid ascorbic PC group with 194 UmL^−1^ for iEPS–1.5 kGy vs. 192 UmL^−1^ for the PC group (Figure 7B). Previous studies reported that low MW EPSs (LMWEPS) exhibit more interesting antioxidant capacities [62,63]. Xu et al. [62] demonstrated that LMWEPS EPSs synthesized by *Bifidobacterium animalis* RH showed stronger antioxidant activity.

#### 3.3.3. Antitumor Activities of nEPS and iEPSs

**Figure 8:**
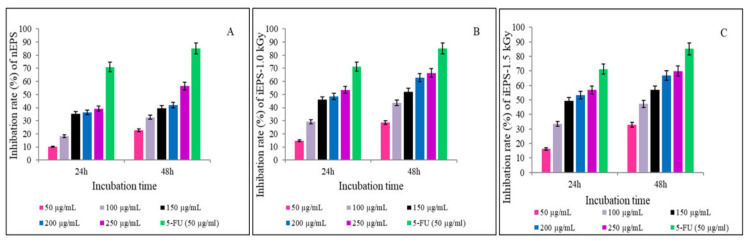
Antitumor effects of (**A**) purified nEPS, (**B**) iEPS–1.0 kGy, and (**C**) iEPS–1.5 kGy on HT 29 cancer cell line. 5-FU was used as positive control. All values were expressed as means ± SD of three replications.

The findings of anti-proliferative capacities of nEPS and iEPSs (1.0 and 1.5 kGy) against the HT-29 cell line of human colon cancer are summarized in Figure 8A–C. Figure 8A demonstrated that the inhibition effects of nEPSagainst the HT-29 cell line significantly increased (*p* < 0.01) as well as the increasing of nEPS concentrations. Similar results were observed with the increasing of treatment period. In fact, at the weakest concentration (50 μg mL^−1^) of nEPS and treatment period of 24 h, the inhibition rate was 10.2 ± 0.1%. However, after treatment with the concentration of 250 μg/mL and an incubation period of 48 h, the anti-proliferative activity was significantly (*p* < 0.01) enhanced (56.8 ± 0.12%) but remained below that observed in the case of the 5-FU positive control (85.2 ± 0.1%). The anti-proliferative effects of iEPSs against the same cancer cell line are shown in Figure 8B,C. We noticed a significant increase (*p* < 0.01) in anti-proliferative activity with increasing the irradiation dose of nEPS. Indeed, the anti-proliferative effect, at the lowest concentration and treatment period of 24 h, increased to 14.8 ± 0.2 and 16.3 ± 0.16% in case of iEPS–1.0 and iEPS–1.5 kGy, respectively against nEPS (10.2 ± 0.1%). Interestingly, at the highest concentration and after 48 h of incubation, the inhibition rates of HT-29 cell line proliferation increased significantly (*p* < 0.01) up to 66 and 69% for iEPS–1.0 and iEPS–1.5 kGy, respectively, and they were similar to the positive control 5-fluorouracil molecule (5-FU) (71.2 ± 0.23% at 24 h incubation period). In comparison to previous research findings, the iEPSs inhibition rate against the HT-29 cell line was three-fold better than that obtained by crude EPS from *L. plantarum*-12 at the same dose of 250 μg mL^−1^ (28.4 ± 1.37) [64] and two-fold better than that of EPS from L. casei SB27 (35.98 ± 0.47) [65]. The irradiation-induced increase in the anti-proliferative activity of iEPSs could be attributed to their low MW as well as to physicochemical changes of the nEPS by radiolysis, as demonstrated above by different analytical tools.

It has been reported that the structural properties, including the MW and type of monomers linkages of the EPS, greatly impact their biological activities and mainly their interactions with proteins [66,67]. Tukenmez et al. [67] investigated the impacts of the MW of lactobacilli-EPS on HT-29 cells inhibition. They demonstrated that the highest anti-proliferative activity in the cells exposed to different MW EPSs (GD2-EPS, E9-EPS, LB63-EPS, and B3-EPS) was 80.7 ± 1.8% obtained with the EPS having the lowest MW (GD2-EPS, 2.4 × 10^3^ Da, at 400 µg/mL). Our findings regarding the effect of MW of nEPS and iEPSs on anti-proliferative activity are comparable to those obtained by Tukenmez et al. [67]; indeed, a maximum inhibition rate of HT-29 cells proliferation (66%) was obtained with iEPS–1.5 kGy having the lowest molecular weight (1.78 × 10^2^ and 1.27 × 10^3^ Da). It has been indicated that low MW EPSs can easily penetrate the membrane barriers of cells and provide greater activity [63]. However, some other studies demonstrated that high MW EPS showed higher rates of inhibition than low MW EPSs [68]. The influence of MW on the anti-tumoral effect of EPS is still controversial [69]. Since high MW EPS are unable to cross the cytoplasmic membrane of cells, this suggests that low MW and high MW EPSs exert their anti-proliferative effects via different mechanisms. The anti-proliferative power of nEPS and iEPSs could be triggered through various mechanisms. In fact, recent studies have shown that EPSs inhibit the proliferation of tumor cells through several strategies including membrane potential breakdown, cell cycle disruption, activating apoptosis signaling, prevention of epidermal growth factor receptor phosphorylation, and expression of tumor-suppressor genes [65,67,70,71].

### 3.4. Genes Implicated in nEPS Biosynthesis

#### 3.4.1. Genomic Analyses of the Enzymes Involved in the Nucleotide Sugars Biosynthesis for nEPS Production

**Figure 9:**
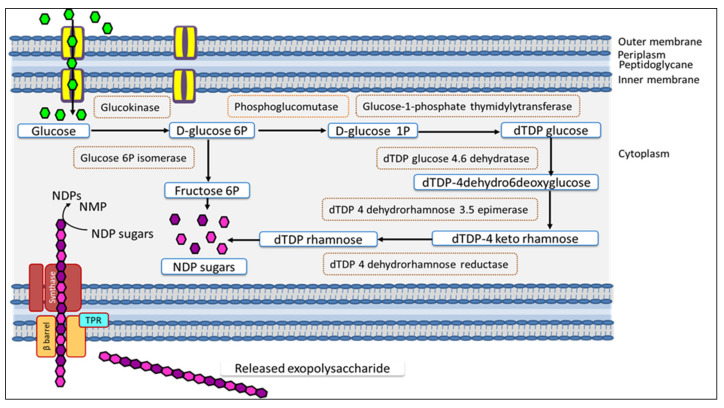
Genes identified for the native exopolysaccharide biosynthesis nEPS in *H. desertis* G11 from the genomic data. Results obtained from tBLASTn analysis and the synthase-dependent pathway for the assembly and transport of nEPS. Details are provided in the Supplementary Data Table S1. 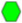: glucose used as carbon source, 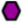: fructose 6P, 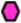: dTDP rhamnose, TPR: tetratricopeptide repeat protein, β-barrel: outer-membrane porin. Synthase complex composed of glycosyltransferase (GT) and co-polymerase.

The mechanism of nEPS synthesis involves three important steps, namely the synthesis of nucleoside diphosphate sugars (NDP sugars), polymerization of the repeat monomers, and then translocation and secretion [12,72]. Since nEPS is an hetero-EPS composed of rhamnose and fructose, genes implicated in the production of dTDP rhamnose and dTDP fructose were mined in the complete genome sequence of *H. desertis* by using the Basic Local Alignment Search Tool (BLAST) against known sequences from the National Center for Biotechnology Information (NCBI) Genbank database (https://www.ncbi.nlm.nih.gov/genbank/, accessed on 10 June 2021). Intracellular production of the nEPS starts with glucose uptake, dTDP rhamnose, and dTDP fructose pathways and then the assembly. The incorporated glucose molecules from the culture medium are converted to dTDP rhamnose and dTDP fructose by enzymatic chemical modifications.All genes involved in these modifications were identified (Figure 9). The glucose is phosphorylated on the sixth carbon through glucokinase (fig|6666666.596377.peg.1088) generating the D-glucose 6P. The D-glucose 6P is converted by phosphoglucomutase (fig|6666666.596377.peg.1957) to D-glucose 1P. The D-glucose 1P is transformed to dTDP glucose using the glucose-1-phosphate thymidylytransferase (fig|6666666.596377.peg.568). The dTDP glucose is dehydrated on the fourth and sixth carbon to dTDP-4dehydro6deoxyglucose using a dTDP glucose 4.6 dehydratase (fig|6666666.596377.peg.570). The dTDP-4dehydro6deoxyglucose is transformed to dTDP-4 keto rhamnose using a dTDP 4 dehydrorhamnose 3,5 epimerase (fig|6666666.596377.peg.567). The dTDP-4 keto rhamnose is converted to dTDP rhamnose using a dTDP 4 dehydrorhamnose reductase (fig|6666666.596377.peg.569). For dTDP fructose, the D-glucose 6P is transformed to fructose 6P through a glucose 6P isomerase encoded by the gene fig 6666666.596377.peg.1116. All these enzymes implicated in the production of NDP sugars from glucose are summarized in Figure 9 and in Table S1 in the Supplementary data.

#### 3.4.2. Genomic Analyses Enzymatic Regulation of the Biosynthesis of nEPS

It is well known that nitrogen has a crucial role in the regulation of EPS biosynthesis. Dalsing and Allen [73] indicated that nitrate reductase is important for EPS production from *R. solanacearum* and; they quantitatively proved the role of nitrogen assimilation in EPS biosynthesis. Genomic analyses of *H. desertis* G11 predicted the presence of genes implicated on the nitrogen regulatory mechanism. Protein-PII uridylyltransferase (EC 2.7.7.59) was encoded by the gene *fig|6666666.596377.peg.1974*. Thus, the signal is transmitted by a dual-component signal transduction mechanism engaging the nitrogen regulatory protein Ntrc encoded by the gene *fig|6666666.596377.peg.704*, which is phosphorylated by the nitrogen regulation protein NtrB (EC 2.7.13.3) encoded by the gene *fig|6666666.596377.peg.705*. Moreover, the gene *fig|370767.3.peg.587* encoding the EPS synthesis ExoD was identified.

#### 3.4.3. Genomic Analyses of Enzymes Involved in the nEPSExportation

Regarding nEPS export outside the cell, genomic analyses suggested the absence of genes encoding for (i) the outer membrane polysaccharide export protein (OPX) and (ii) the polysaccharide co-polymerase protein (PCP), which are both essential for the Wzx/Wz-dependent pathway and the ABC transporter-dependent pathway. According to these data, we can infer that *H desertis* does not use either of these two systems for the production ofnEPS. Thus, it is proposed that *H. desertis* uses the synthase-dependent pathway to realize the assemblage and the transport of nEPS (Figure 9). The synthase-dependent pathway requires a core of proteins composed of (i) an inner-membrane (IM)-embedded polysaccharide synthase, (ii) a (TPR) periplasmic tetratricopeptide repeat protein, and (iii) an(β-barrel) outer-membrane (OM) β-barrel porin. It is relevant to mention that synthase-dependent systems could be post-translationally regulated by c-di-GMP (bacterial second messenger bis-(3′–5′)-cyclic dimeric guanosine monophosphate) [74]. The protein core already mentioned was identified in the genome of *H.desertis*. Three proteins that mediate the formation and insertion of β-barrel proteins into the OM were detected and identified: BamB, BamD, and BamE encoded by genes fig|6666666.596377.peg.3383, fig|6666666.596377.peg.890, and fig|6666666.596377.peg.654, respectively. The tetratricopeptide repeat (TPR) family protein is predicted to be encoded by the genefig|6666666.596377.peg.598. Additionally, c-di-GMP involved in the synthase-dependent pathway was identified in the genome of the strain G11 and is encoded by the gene fig|6666666.596377.peg.2793. Moreover, the genome of *H. desertis* harbored various c-di-GMP synthesized by diguanylate cyclases (DGCs) as well as the integration host factor (IHF), which play a key role in the regulation of EPS biosynthesis. All these data are summarized in Table S1 (Supplementary data) and Figure 9.

There is little published data on genomic analyses on EPS biosynthesis by *Halomonas* strains. In concordance with our study demonstrating for the first time that *H. desertis* uses a synthase-dependent pathway to produce nEPS, Arco et al. [75] identified four genes, *epsA*, *epsB*, *epsC,* and *epsJ*. The latter is a wzx homolog. They demonstrated their role in mauran biosynthesis and indicated that *H. maura* S-30 uses the Wzx/Wzy-dependent pathway to realize the polymerization and the transport of mauran [75]. In addition, recently, genomic analyses undertaken by Athmica et al. [43] reported that *H. malpeensis* strain YU-PRIM-29T has an ABC transporter-dependent pathway to synthesize and export EPSs outside the cell. These findings suggest that *Halomonas* strains, such as *Lactobacillus*, could produce EPS via different biosynthesis pathways.

## 4. Conclusions

In this work, iEPSs obtained by gamma irradiation of nEPS exhibited superior antioxidant activities in vitro. In addition tothe interesting properties that nEPS naturally possesses, gamma irradiation endows this macromolecule with other new functional characteristics such as low molecular weight, number of reducing ends, functional groups, and smaller particle sizes. iEPSs significantly ameliorated HF diet-induced oxidative stress with particular enhancements of antioxidant enzymes levels and a decrease in the content of MDA. Moreover, iEPSs’ anti-proliferative activity against HT-29 cells increased significantly and was nearly to the positive control. From a genomic point of view, the genome of *H. desertis* harbored genes involved in biosynthesis pathways and export mechanisms of nEPS. The strain G11 was demonstrated as the first *Halmonas* species that follows the synthase-dependent pathway to execute the assembly and the transport of the nEPS. Further research should be carried out to optimize the gamma irradiation dose in order to obtain oligomers with desired functional properties.

## Figures and Tables

**Table 1:** Schematic representation of the experimental protocol for different groups. Animals were randomized into five groups (*n* = 10 mice in each group; NC group: normal control group; PC group: positive control group; nEPS group: native EPS group; iEPS–1.0 kGy group: 1 kGy gamma-irradiated EPS group; iEPS–1.5 kGy group: 1.5 kGy gamma-irradiated EPS group).

**Acclimatization** **Period** **(10 days)**	**Different groups**	**Treatment period** (14 days)	**Blood Collection** **(One day After Treatment Period)**
		**Orally administration of 0.5 mL** (once a day/mouse) of	
	NC group	saline solution (0.9%)	
	PC group	ascorbic acid (400 mg kg^−1^ body weight)	
	nEPS group	native EPS (400 mg kg^−1^body weight)	
	iEPS–1.0 kGy group	1 kGy irradiated EPS (400 mg kg^−1^ body weight)	
	iEPS–1.5 kGy group	1.5 kGy irradiated EPS (400 mg kg^−1^ body weight)	
**−10 0**		14th	15th

**Table 2:** Characteristics of EPSs produced by *Halomonas* strains (* among the total carbohydrates 50% were uronic acid, T: It's part of the strain code).

*Halomonas*	Strain	EPS Composition				MM (Daltons)	References
		Carbohydrates	Proteins	UronicAcid	Sulfate		
*H. eurihalina*	F2-7	37	7.5	ND	11.2	ND	[49]
	A1-12 ^T^	30.9	2.07	ND	1.1	5.3 × 10^4^	
*H. maura*	S-30	65	2.5	ND	6.5	4.7 × 10^6^	[46]
*H. ventosae*	A1-16	30.8	3.95	ND	0.7	5.2 × 10^4^	[47]
*H. anticarinesis*	FP36	33.7	0.4	ND	1.5	2 × 10^4^	
*H. almeriensis*	M8 ^T^	30.5	1.1	ND	1.4	1.5 × 10^4^ 6.3 × 10^6^	[45]
*H. nitroreducens*	WB1	68.9	2.2	ND	ND	1.3 × 10^3^ 5.2 × 10^6^	[50]
*Halomonas* sp.	MCTG39a	17.2	36.4	ND	ND	2.61 × 10^5^	[51]
*H. smyrnensis*	K2	34.47	0.75	1.01	ND	3.96 × 10^5^	[52]
*H. saliphila*	LCB169 ^T^	97.6 ± 1.5	0	ND	0	5.13 × 10^4^	[53]
*H. malpeensis*	YU-PRIM-29 ^T^	76	5	50 *	ND	ND	[43]
*H. Desertis*	G11	61	0	17.5	10.2	nEPS: 2.42 × 10^4^ iEPSs: 1.27 × 10^3^ 1.78 × 10^2^	This work

## Data Availability

Not applicable.

## Supplementary Materials

The following are available online at https://www.mdpi.com/article/10.3390/polym13213798/s1, Figure S1: Standard curves: (A) Glucose standard curve (B) glucuronic acid standard curve and (C) Potas-sium sulfate standard curve plotted to estimate total carbohydrate, uronic acid and sulfate con-tents in nEPS, respectively, Table S1: Genes implicated in nEPS production by H. desertis G11.
