# Supplementary material for: Effect of Gamma Irradiation on Enhanced Biological Activities of Exopolysaccharide from Halomonas desertis G11: Biochemical and Genomic Insights
Source: Polymers (Basel). 2021 Nov 2;13(21):3798. doi: 10.3390/polym13213798 (PMC8588121; doi:10.3390/polym13213798)
Supplement: Supplementary file 1 [file polymers-13-03798-s001.zip › polymers-1394306-supplementary.pdf]

Table S1

Protein function		Gene code	Gene name	Protein sequences
Nucleotide sugarbiosynthesis	Dtdp rhamnose	fig|6666666.596377.peg.1088	Glucokinase	MRPALIGDIGGTNARLALVTPGDITPHDIINLPCADYPGVIEAIQDY<br>LTRVGAAGDNAPREACLAFACPVHAERVKMTNNHWDFRKSEVR<br>ETLNLSLFKVINDFTAQALGVPHVTADDLVAVQAGDGQAHSTRL<br>VIGPGTGLGVAGVFPGQHAWIPLPTEGGHVTFAPTDDTERALLDV<br>FLQHHKRVSVERILCGQGLLELYQAHCALDDQAPRCTSPAEVTQ<br>AANQGDPIATATLLRFLKILGDVCGDATLTMGARGGVYLCGGILP<br>RLLDWLPKSQLRDGFVNKGRMGAYNADIPVWVVTHPWTGLLG<br>AAEALHNEEVF
		fig|6666666.596377.peg.1957	Phosphoglucomutase	MTTPTIPASIFRAYDIRGIVDDTLTEDTVEWIGRAIGSAAATRGEST<br>VVVARDGRLSGPRLQAALMRGLNAAGRDVIDIGMVPTPVLYFAT<br>HILDGTRSGVMVTGSHNPPDYNGFKIVLDGDTLSGDAITALYERL<br>QSGDLTQGEGRIRQEDVRDAYLTRILGDVTINRPIKAVVDCGNG<br>VAGELGPQLIERLGVDTIPLFDEIDGTFPNHHPDPGKPENLQDLIRT<br>VQETGADIGLAFDGDGDRLGVITPRGRLIYPDHLLMAFATDMLSR<br>NPGAKVIFDVKCTGNLVKVISDAGGEPEMWRTGHSLIKARMKET<br>GAQLAGEMSGHIFFKERWYGFDDGLYAAARLVEILANYAGDAD<br>AYFDSFPQDIGTPEINITVTDSNKFNLVDKLAREGDFGDGIKTTLD<br>GIRVDYPDGWGLCRASNTTPVLVLRFEGKNDAALARIKAQFANA<br>LKDVDPALTLPQA

		fig|6666666.596377.peg.568	Glucose-1-phosphate thymidylytransferase	MKGIILAGGSGTRLYPITRGISKQLLPVYDKPMIYYPLSVLMLAGIRDILVITTPEDQDGFQRLLSNGSQWGINLTYAAQPSPDGLAQAFIIGEKFIGGDSVCLVLGDNIYYGQGLSRMLQAAATRDKGATVFGYQVNDPERFGVVEFDDNLRAISIEEKPANPKSDYAVTGLYFYDNDVVDIAKQVEPSERGELEITSINQAYLERGDLNVELLGRGFAWLDTGTFDSLHDAASFIETLEKRQGLKIACLEEVAYRMGFIGEKELLAEAEKLKKNSYGTYLKKLAKTANKRAL
		fig|6666666.596377.peg.570	dTDP glucose 4.6 dehydratase	MKLLITGGAGFIGSAVIRHIIRNTEDSVVNVDKLTYAGNLESLAAASDNERYIFEQVDINNRVDLDRVFREQQPDAVMHLAAESHVDRSIDGPADFIETNIVGTYTLLEAARYYWQGLGHERQQVFRFHHISTDEVYGDLPHPADTTNSAHYLFTETTPYAPSSPYSASKASSDHLVRAWRRTYGLPTLITNCSNNYGPYHFPEKLIPLMILNALEGKPLPVYGKGDQIRDWLYVEDHARALYKVITEGKVGETYNIGGNNEKQNIEVVLSLCDILQELYPQSTPYRELINYVPDRPGHDMRYAIDASKIQKELGWQPQETFDTGIRKTVQWYLDHLDWCKRVQDGSYQRERMGFNK
		fig|6666666.596377.peg.567	dTDP 4 dehydrorhamnose 3,5 epimerase	MKIIETRIPDVKIIEPKVFGDERGFFMETWNEKAFREAGINATFVQDNHSRSVKNTLRGLHYQVKQPQGKLVRVTRGEVLDVVVDLRLESPTFGRWVGEYLSEDNNLMLWVPPRFAHGFLVVSDEADFQYKCTDFYAPEYERSIVWDDPDISIEWGVSEVDKLRLSAKDMMGQSFDAAKTELLISS

		fig|6666666.596377.peg.569	dTDP 4 dehydrorhamnose reductase	MKILLLGKTGQVGFELHRSLSSLGTVIAPGRAELDLHKEHAVANY<br>LAYTKPSMIVNAAAWTAVDAAEGKKNEAERLNTGLPAQLAQYA<br>AANSARLFHYSSDYVYPGNGEMPWKENSDTGPLSHYGKTKLKG<br>DEAIEQSSADYVIFRTSWVYSARGSNFMKTMLRLAKSKSELSIVA<br>DQIGAPTPARLIAQVTTLAIHRQLRKGLYHLVPKGETSWYGFAQE<br>IFLLAQKNGEQLTISPDNVYPIPTSDYPTPATRPLNSRMEATKLET<br>ALSIQLPDWQSQLELTLCEYLEK
	**Dtdp**	fig|6666666.596377.peg.1269	Glucose-6-phosphate isomerase (EC 5.3.1.9)	MAASSRTPPDSLPAWQTLKQHAQALKHVHLKNLFGNDPGRWTH<br>FTRQVAGLTLDLSKQRWDDDTLEHLLALAEEAGVPGAIEALLSG<br>KRVNVSENRPALHTALRLPPGASLDVEGEDVAAAVHESLTQMER<br>LVARLHAGQWRGATGKPIRHVVNLGVGGSDLGPQMVTHALAD<br>YRPDDIHPVEVHFASTMDGSQLADYLTRFNPETTLFVLSSKSFTTI<br>DTLSNANTARDWLIGRLSKHGELPSANPVSEELIIRQHFIGVSASP<br>DKMSEWGITPDHQLMFWEWVGGRYSLWGTIGLPIALVVGMENF<br>RELLAGAHAMDRHFQEAPLAENLPVLLGLAGIWNVNFLDIRAHSI<br>LPYDGRLEYFAAYLEQLEMESNGKSVTGQGQAVNYSTCPVLWG<br>QLGPNAQHAFYQLLHQGTQPVVCDFIAPLKRYDEVEDPDTRRHL<br>KSQHRLALANCFAQSRVLMLGDDAIDDDGPRPAHKRYRGN<br>QPSTTVLLDKLTPATLGSLIALYEHKVFVQAVIWDINPFDQWGVE<br>LGKQIATDTRKIIDHQGDLARLDASSRGLIEAFWAAEQE
**Regulationof biosynthesis**		fig 6666666.596377.peg.1974	Protein-PII uridylyltransferase	MLLHHYRFEPDTTLFDLELFRTELAGSRSPIAPFKAALGEIQTRLD<br>ERFRAGADIRDLVRGRAWYLDQLLAIAWEQHDWPDDGVALVAV<br>GGYGRGELHPHSDIDLLLLLEHDDDTPYREPLTAFITFLWDIGLEI<br>GHSVRSLNDCEREAAADVTVITNLLESRLIAGPEALRERMRERLN<br>ADHVWPADRFFEAKWQEQIARHYRYNNSEYHLEPNLKSSPGGLR<br>DIQMIGWVAKRHFGTEEYTDIVANGFMNDAELRILSQGQAFLWQ<br>VRYALHMLTGRAEDRLLFDHQRTIAEMFGFRDTPEGLAVEQFMK<br>RYYRHVTALAGLNDMLLQHFDEVILRGKEALETVKLNERFETKG<br>GYIQVRSRNLFREQPSAMLELFLLMAKHPEIEGVRADTIRLIRDHR<br>HQIDDHYREDPHHQRLFMAIMRAPGNVPRQLRRMNRYGILGKYL<br>PEFGRAVGLMQHDLFHIYTVDAHTLRLLKFLHGFRKPEAKDDFP<br>VAATLMQQLPKLDLLWIAGLFHDIGKGRGGDHSEIGARDVEQFC

			QRHHVPQHDTNLVSWLVEHHLLMSMTAQKRDISDPDVIRDFAM<br>EVRNETRLDYLYVLTVADINATNPTLWNGWRASLLRQLHAETKR<br>ALRRGLKNPPDRDDWVRETRTEARSLLQTIGVDETDIDRLWDSL<br>GEDYFLQYAPSEIVWQTQGILAHQPSPLPLVLISAPTADMTEGGT<br>KVFIHTRSVDDLFAATAAAMEQLGLSIHDARIATSNNDWTLNTFI<br>VLDNQGQPIRDPARIEEMRQHLVEELDDPDDYPDIVTRHTPRQLK<br>HFRVATEVLIEQDPANDRTLLELSAPDRPGLLARVGRIFMEQDIAL<br>SAAKIATLGERVEDVFFITTKAGEPLTDPDRQQQLRERLIEVLGV
	fig|6666666.596377.peg.704	Ntrc	MTEPARNDVARVVIVDDDRAIRWVLERALAQPDLEVECIERADT<br>ALSRLLESPPDVLVTDIRMPGIDGLDLMARVREAHPDLPVIVMTA<br>HSDLDSAVASYQGGAFEYLPKPFDVDEALALVRRAIAHARERQR<br>PVTVPEGLNAEIIGEAPAMQEVFRAIGRLSHSHITVLINGESGTGK<br>ERVAEALHQHSPRQGKPFIALNMAAIPRDLIESELFGHEKGAFTG<br>ATSQRQGRFEQANGGTLFLDEIGDMPAETQTRLLRVLADGEFYR<br>VGGHTPTKVDVRIIAATHQNLESLVDDGRFREDLFHRLNVIRIHLP<br>KLAERREDIPRLTSHFLAEAAKELSTDVKVLTPEAEAHLTRLPWP<br>GNVRQLENICRWLTVMASGREVLVEDLPAELRSPSASESSAHGD<br>WRSAFRDWADHALAEGHTHLLEEAVPDFERILIETALKHTGGRK<br>GEAAELLGWGRNTLTRKLKTLLPALADE
	fig|6666666.596377.peg.705	NtrB	MQDTTMHQRLLEHLTTAVLLLDGRLNVRWMNPAAEALFAVSLS<br>RVKGMSLDGMLGGDESIDEVLAKARDAFHPFTQREARITPLNSDP<br>LTVDYTVTPLSDMELLLEVEPRDRLMQISREEALTTRQETIKVLA<br>RGLAHEVKNPLGGIRGAAQLLERDLDNPALREFTHIIVQEVDRLR<br>DMVDSMLGPNYIVKHEPVNIHKVLERVRSLLIAEHPWVDIQRDY<br>DPSLPELFGDEAQMIQAVLNVARNAVQAMGDAETPTPTLVLRTR<br>ARRQFTLGAERHRLVSEVGIIDNGPGIPGTLQETLFYPMVSGRAE<br>GSGLGLSIAQSILHQHQGLIECDSRPGRTEFRLLIPLVINVTGEAS
	fig|370767.3.peg.587	Exopolysaccharide synthesis, ExoD	MDDRREGSTLMDLIASLERMEQDAQRVSVDDVVHAVGRRSFGP<br>LLLVTGLITLAPIIGDIPGMPTLMALLVLLVSVQLLVGRETFWLPG<br>WMLKRSISRDKFDKGIYYLKKPARWIDGLLRVRLPWLTGYIGIRV<br>TAVVCLMIALAMPPMEFIPFSANGAGLALSLLGLGLVARDGAAL<br>LLGVALFGVTCTLILVGLL

**Regulation of the EPS excretion**	fig|6666666.596377.peg.2793	PilZ	MATQKALSLTVPDVPTLLSAYMPFLDRGGMFIPTRGYYDLGQTV<br>YLLLTLPGESERLSLSGEVIWVSPDGVTGRRMPGIGIHFNAQDYR<br>VRDRIETLLAGQLDKAAPSFTL
	fig|6666666.596377.peg.598	Tetratricopeptide	MLLPKRSTIVATLAGLTLTALMIPSAWALDDEAQAAKKEEGMRL<br>WGIHQWEKMQPPLETAAESGDVEAMYYLGEANRLLSRGLSQAA<br>LDWYHQAAQHGDPYAMLRLFDGGACELGDVCPENGDDWPQAA<br>LELTLPKAEAGDPEAMAALYDIYFYVKDPDEDEAMKWLRRAAE<br>AGQVESMNLLGKIARNDEESYANDTERLEAAEVWFRKAAEAGY<br>APAMNNLAAVLSNLERNEEAWEWMATASEAGHINGRRWVAAC<br>NIVHEEQGRDLCRSAAKPDPAKGWAIVLATKQEVPNTYSTATLE<br>RYRDKISPEQRKEGEEIVDEWLNREPPLSYFPEKFGP
	fig|6666666.596377.peg.3383	beta-barrel BamB	MTPLFSTSMFSRSTFSKRTFSTPVMRVALGAAALALLAGCASKSE<br>PAYTPKELKSFEATSTLETLWQEDVGDGLGRARYPIAPAREGDN<br>VFAADAQGVVMSFSADDGEERWEVDLDTPISSALTAIAGQVYLG<br>TRNGEVISLDQSDGSVNWRSRVSSEVLAAPQANPELLVVQSIDGQ<br>VTALDRASGDERWVFSSSLPSLTLRGTGTPMVIEPVSFVGLANGR<br>LTTIDNRNGQPLWDMQIATPQGRSEVERLVDLAGQPVLSREGRLF<br>VTSYNGQLVALEATRGNVIWERELSSRHTPLLVGDLLFVVTDDS<br>HVVAIDSTNGQEVWRNDALEDRWLTAPAFADGRVVVGDFEGYV<br>HLIDAREGELVGRTEVDSSGISVPAVTEGDVIHVLANDGHFETLE<br>VSP
	fig|6666666.596377.peg.890	beta-barrel BamD	MRVFSAANRFGVFALSFALLAGCASNGNNDTAYDEDEYAGVAE<br>RELYERARDALDANRFNIAVERLEALDTRYPFGEHAEQAQLELIY<br>AYYENGNWEEARAAASRFIRLHPDHPQVDYAYYLRGLSAWQAG<br>RFSLERLRLIDISKRDLGATRDAYSDFRELIQRYPQSEYAPDAQQR<br>IVYLRELLARHELHVADYYLRRGAYLAAVERGRWVVEKYPESN<br>ATHDALATMVEGYLGLEMDDRANEVLAVLRDNAPNHDQLEGN<br>RFVPKHID
	fig|6666666.596377.peg.654	beta-barrel BamE	MQKLTRIITLSVSIAVISGCSYVGVYKRDIPQGNLVTEEMVDQLQP<br>GMTQEQVTYVMGRPLLEAPFDASEWDYVFRLDKAYAGVEQRR<br>VTLTFDDQGRLANIDQEGDFSKDIPLEADSTGGPAPDTTDPTEAI<br>PNEPRQNTTPSTTN

Figure S1 standard curves: (A) Glucose standard curve (B) glucuronic acid standard curve and (C) Potassium sulfate standard curve plotted to estimate total carbohydrate, uronic acid and sulfate contents in CS-EPS, respectively

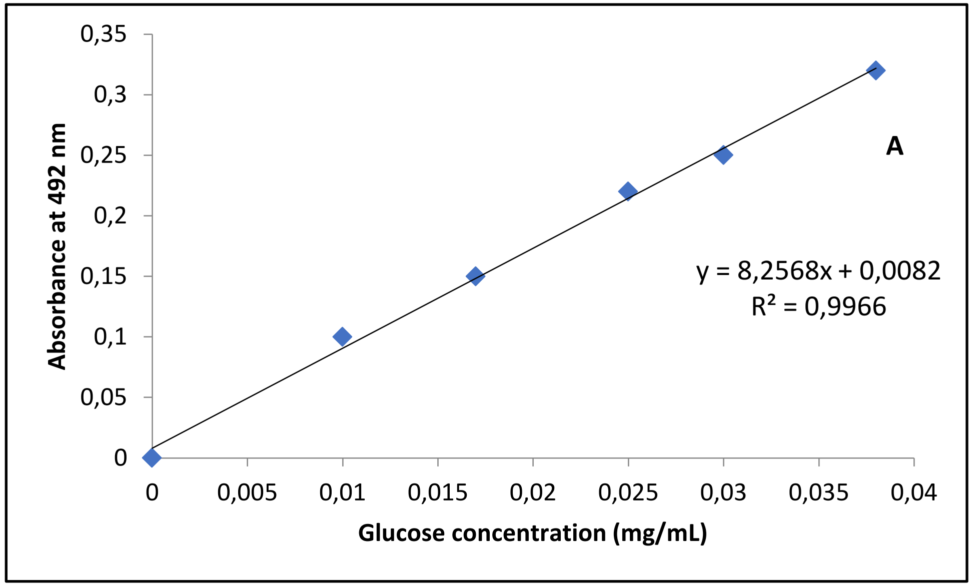

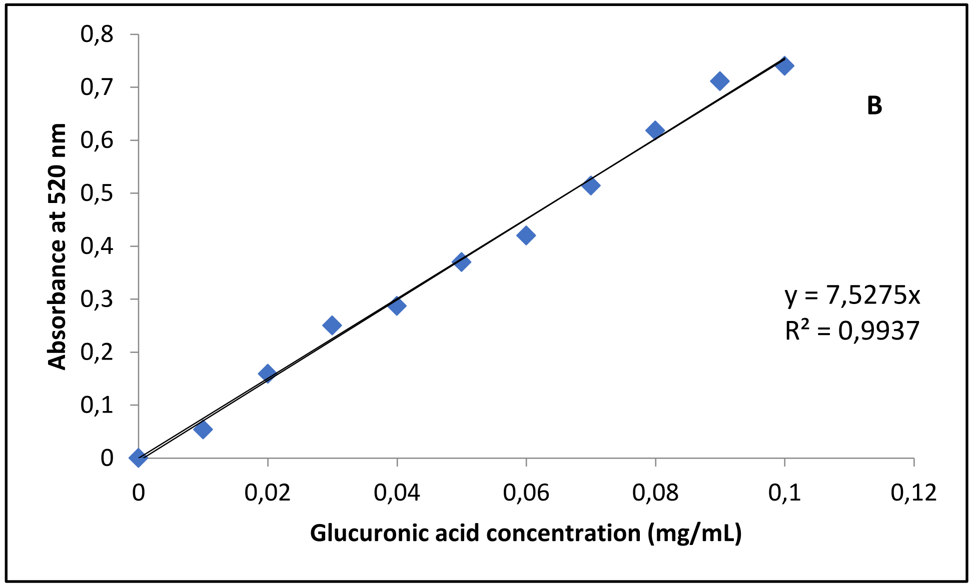

B
y = 7,5275x
R² = 0,9937
Absorbance at 520 nm
0
0,1
0,2
0,3
0,4
0,5
0,6
0,7
0,8
0
0,02
0,04
0,06
0,08
0,1
0,12
Glucuronic acid concentration (mg/mL)

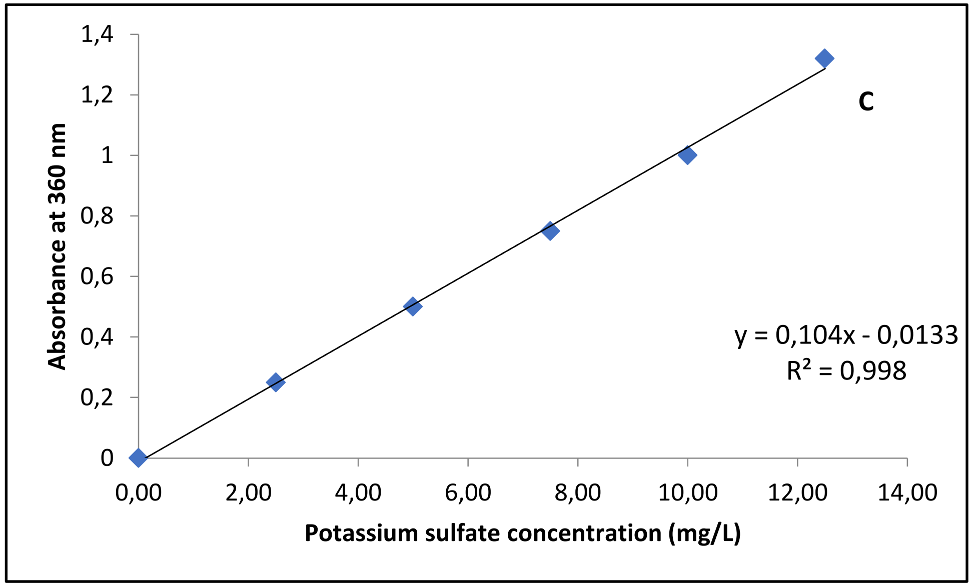

1,4
1,2
1
0,8
0,6
0,4
0,2
0
Absorbance at 360 nm
C
y = 0,104x - 0,0133
R² = 0,998
0,00
2,00
4,00
6,00
8,00
10,00
12,00
14,00
Potassium sulfate concentration (mg/L)
